# A novel recombinant 6Aβ15-THc-C chimeric vaccine (rCV02) mitigates Alzheimer’s disease-like pathology, cognitive decline and synaptic loss in aged 3 × Tg-AD mice

**DOI:** 10.1038/srep27175

**Published:** 2016-06-03

**Authors:** Yun-Zhou Yu, Si Liu, Hai-Chao Wang, Dan-Yang Shi, Qing Xu, Xiao-Wei Zhou, Zhi-Wei Sun, Pei-Tang Huang

**Affiliations:** 1 Beijing Institute of Biotechnology, Beijing 100071, China; 2 Institute of Life Science and Biotechnology, Beijing Jiaotong University, Beijing 100044, China

## Abstract

Alzheimer’s disease (AD) is a neurodegenerative disorder that impairs memory and cognition. Targeting amyloid-β (Aβ) may be currently the most promising immunotherapeutic strategy for AD. In this study, a recombinant chimeric 6Aβ15-THc-C immunogen was formulated with alum adjuvant as a novel Aβ B-cell epitope candidate vaccine (rCV02) for AD. We examined its efficacy in preventing the cognitive deficit and synaptic impairment in 3 × Tg-AD mice. Using a toxin-derived carrier protein, the rCV02 vaccine elicited robust Aβ-specific antibodies that markedly reduced AD-like pathology and improved behavioral performance in 3 × Tg-AD mice. Along with the behavioral improvement in aged 3 × Tg-AD mice, rCV02 significantly decreased calpain activation concurrent with reduced soluble Aβ or oligomeric forms of Aβ, probably by preventing dynamin 1 and PSD-95 degradation. Our data support the hypothesis that reducing Aβ levels in rCV02-immunized AD mice increases the levels of presynaptic dynamin 1 and postsynaptic PSD-95 allowing functional recovery of cognition. In conclusion, this novel and highly immunogenic rCV02 shows promise as a new candidate prophylactic vaccine for AD and may be useful for generating rapid and strong Aβ-specific antibodies in AD patients with pre-existing memory Th cells generated after immunization with conventional tetanus toxoid vaccine.

Alzheimer’s disease (AD) is characterized by senile plaques (SPs) and neurofibrillary tangles (NFTs). The onset and progression of AD is thought to be caused by the production and accumulation of excessive amyloid-β (Aβ) in the brain, which results in amyloid plaque deposition as a defining pathological hallmark, and ultimately leads to neuron loss, cognitive decline and brain atrophy^1^,^2^. Human Aβ-directed active and passive immunization can effectively clear the cerebral Aβ load in various AD mouse models^3^,^4^,^5^ and human AD patients^6^,^7^,^8^,^9^. Furthermore, immunotherapeutic reduction of Aβ in the brain ameliorates AD-like behavioral symptoms in AD model mice and, in humans, immunotherapy with a monoclonal antibody directed at the mid-region of Aβ (Solanezumab) has also shown some beneficial cognitive effects in mildly affected AD patients^10^. Therefore, the removal or lowering of Aβ from the brain in patients with very early AD pathology or even in presymptomatic subjects could be an effective therapeutic measure; obviously, a safe active vaccine might be beneficial for such preventive treatments of AD^11^,^12^.

Synapse loss occurs early in AD and accompanies Aβ accumulation; therefore, these characteristics are considered the best neuropathological correlates of cognitive decline^13^,^14^,^15^,^16^. Some therapeutic strategies for AD attenuate synaptic dysfunction and improve cognitive behavior in AD models^17^,^18^,^19^,^20^,^21^,^22^,^23^,^24^. Given the remarkable recovery of cognition in AD models of targeted-Aβ immunotherapy, it is necessary to determine the molecular correlations associated with improvement. A recombinant chimeric 6Aβ15-THc-C immunogen developed as protein vaccine for AD generated a robust anti-Aβ42 antibody response, and attenuated Aβ pathology and cognitive deficits in the PDAPP^V717I^ mouse model^25^. However, the potential of this treatment to rescue synaptic dysfunction in preclinical models of AD remains to be clarified. In this study, this recombinant chimeric 6Aβ15-THc-C immunogen was formulated with alum adjuvant as a novel Aβ B-cell epitope candidate vaccine (rCV02). We performed a comprehensive evaluation of its efficacy for the prevention of the cognitive deficit and synaptic impairment in 3 × Tg-AD mice. Furthermore, we sought to determine the molecular correlations between the recovery of cognition and the improvement of synaptic functions. Moreover, the immune mechanism associated with rCV02 vaccination with the aid of a toxin-derived carrier was defined in 3 × Tg-AD mice.

## Results

### The immunogenicity of rCV02 in 3 × Tg-AD mice

**Figure 1:**
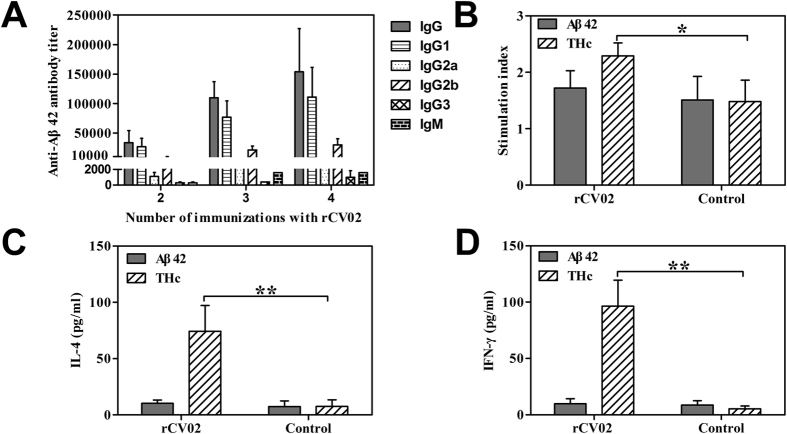
rCV02 induces strong Th2-polarized Aβ-specific humoral immune responses and activates proliferation of THc-specific T cells in 3 × Tg-AD mice. (**A**) Titer of anti-Aβ42 antibodies in 3 × Tg-AD mice immunized with rCV02. The titer was determined for the indicated IgM and IgG isotypes (IgG1, IgG2a, IgG2b, and IgG3). (**B**) T cell proliferation in rCV02-immunized mice. Splenocytes were harvested from rCV02-immunized and control mice and restimulated *in vitro* with 10 μg/mL Aβ42 and THc. Cytokine production from splenocytes was used as a surrogate marker of Th1 (IFN-γ; (E)) and Th2 (IL-4; (**D**)) bias in the immune response to rCV02. IL-4 and IFN-γ levels were measured by ELISA. Data represent the mean ± SD (n = 8). Statistically significant differences were determined by Student’s *t*-test. * *p* < 0.05, ** *p* < 0.01, compared with the control group (3 × Tg-AD).

To evaluate the immune response to the rCV02, the humoral and cellular immune responses were analyzed in experimental and control 3 × Tg-AD mice. As shown in Fig. 1A, high levels of Aβ-specific IgG antibodies were induced in the rCV02-immunized mice following multiple immunizations (2, 3, or 4). Lymphocyte proliferative responses showed that rCV02 induced THc-specific responses, but not Aβ-specific T cell immunity in immunized mice (Fig. 1B, *p* < 0.05) and significantly increased IL-4 (Th2) or IFN-γ (Th1) cytokine levels induced by THc but not by Aβ42 (Fig. 1C,D, *p* < 0.01). Furthermore, immunization with rCV02 resulted mainly in IgG1 and IgG2b isotypes, which are associated with non-inflammatory Th2 responses. These results indicate that the rCV02 can induce robust anti-inflammatory Th2-polarized immune responses without activating self-reactive Aβ42-specific T cells.

**Figure 2:**
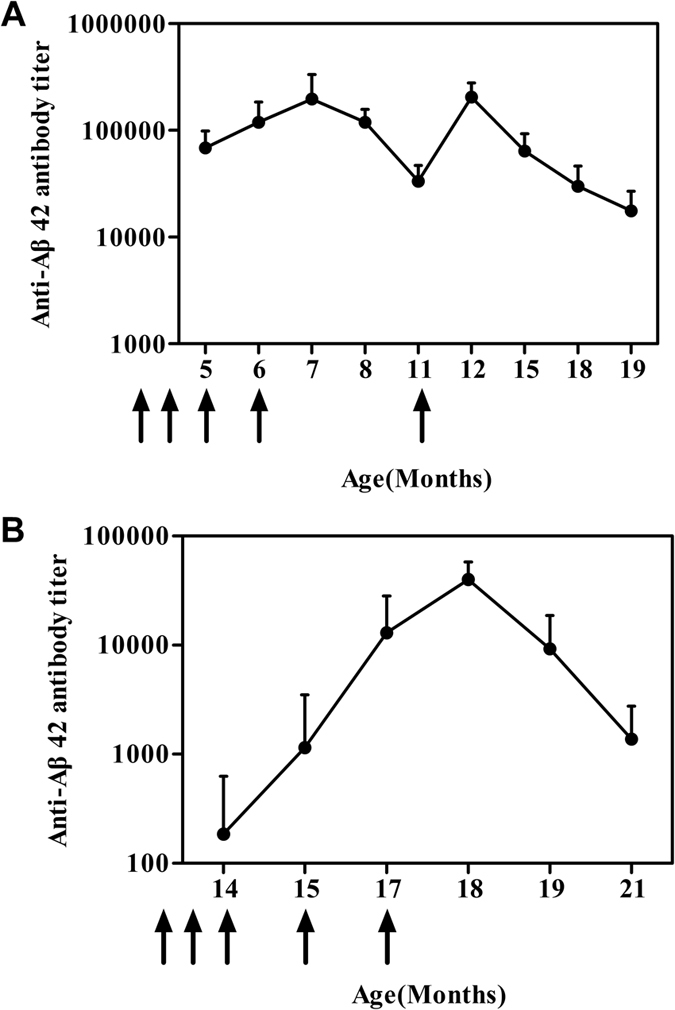
Duration of rCV02-induced immunity in 3 × Tg-AD mice following prophylactic or therapeutic administration. Serum samples from 3 × Tg-AD mice immunized with rCV02 were collected at the time-points indicated (Supplementary Figure S1) and the anti-Aβ42 IgG titers were analyzed by ELISA. Serum samples from individual mice were assayed, and the geometric mean titer (GMT) was calculated for each group (n = 8). The serum antibody titer was monitored for 14 months for the prophylactic treatment (**A**) and 7 months for the therapeutic treatment (**B**). The x-axis indicates the age of the mice at the time of the blood sampling (months). The solid arrows indicate the time of rCV02 vaccine administration.

Having demonstrated that rCV02 elicited a robust immune response, we tested its immunogenicity in prophylactic and therapeutic treatment models (supplementary Figure S1). As a prophylactic treatment, immunization with rCV02 generated a strong Aβ-specific antibody response after two immunizations; all antibody titers were further increased following a booster (Fig. 2A). The antibody levels remained high over a 7-month period after the last booster (five immunizations).

As a therapeutic treatment, rCV02 generated a relatively low antibody response after the initial three immunizations, while the antibody response was markedly augmented by two boosters. However, the high level of Aβ-specific IgG antibodies could not be sustained over a 4-month period after the last booster (Fig. 2B).

### Immunization with rCV02 restores cognitive memory in aged 3 × Tg-AD mice

**Figure 3:**
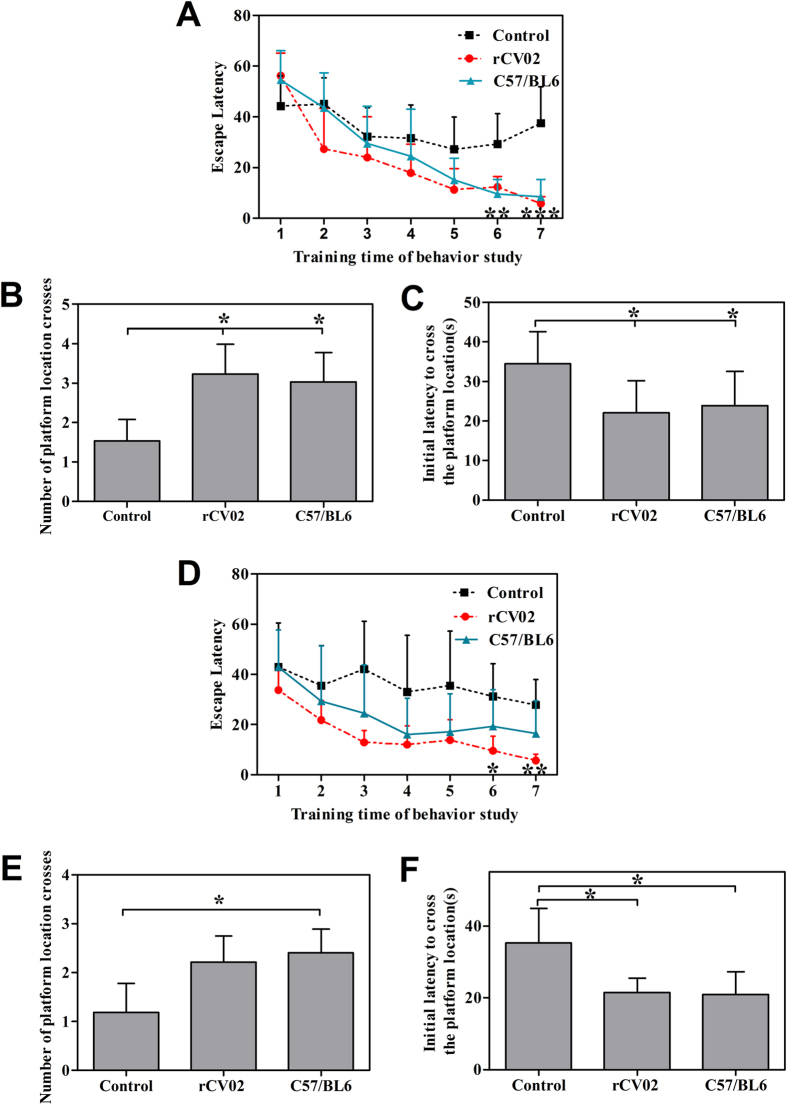
Vaccination with rCV02 improves cognitive performance in aged 3 × Tg-AD mice following prophylactic and therapeutic administration. 3 × Tg-AD mice were immunized with rCV02 at 3 months (prophylactic; panels (**A–C**) or 12 months (therapeutic; panels **D–F**) of age and subjected to the Morris water maze test at 19 months and 21 months of age, respectively. Animals were tested daily for 7 days. (**A,D**) The mean escape latency (time) to reach the platform on days 1–7 of the water test during the training trials. (**B,E**) The mean number of platform location crosses during the probe trial after the last training trial. (**C,F**) Initial latency (time) to reach the platform location in probe trials after the last training trial. Data represent the mean ± SD for each group (n = 6–8). Statistically significant differences were determined by ANOVA. *p < 0.05, **p < 0.01, ***p < 0.001, compared with the control group (3 × Tg-AD).

The spatial learning and memory abilities of 3 × Tg-AD and wild-type (C57/BL6) mice were evaluated using the Morris water maze test. In the prophylactic treatment, the latency to reach the platform for the rCV02-immunized AD mice was significantly decreased on days 6–7 of training (Fig. 3A, *p* < 0.01 and 0.001, compared to the control AD mice) as age-matched non-transgenic C57/BL6. Notably, a decrease trend in this latency was similar to a group of non-transgenic mice of the same age. In the probe trials for spatial memory ability, significantly shorter initial latency to reach the platform and significant increased number of correct platform location crosses were observed in the rCV02-immunized AD mice when compared to the control AD mice (*p* < 0.05, Fig. 3B,C).

In the therapeutic treatment, the rCV02-immunized AD mice also showed significantly shorter escape latencies on days 6–7 compared with the control AD mice (*p* < 0.05 and *p* < 0.01, Fig. 3D). In spatial memory testing, the rCV02-immunized AD mice had a significantly shorter initial latency to reach the platform, and a decrease in the number of correct platform location crosses was observed, while the effect was not statistically significant (Fig. 3E,F). These data indicate that immunization with rCV02 generates effective prophylactic or therapeutic effects on the cognitive memory of aged 3 × Tg-AD mice and restores their cognitive ability.

### Immunization with rCV02 mitigates the development of AD-like pathology in aged 3 × Tg-AD mice

**Figure 4:**
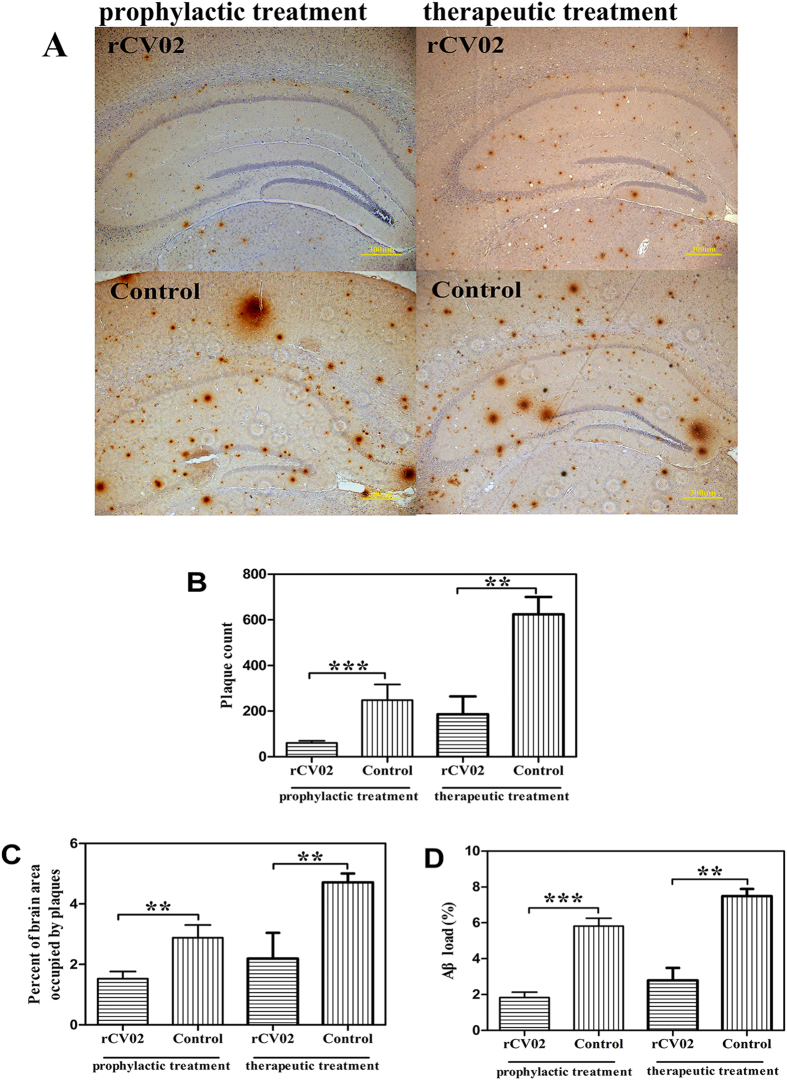
Vaccination with rCV02 significantly reduced the Aβ plaques in brains in aged 3 × Tg-AD mice. 3 × Tg-AD mice were immunized with rCV02 at 3 months (prophylactic) or 12 months (therapeutic) of age. The brains were collected for evaluation at 19 months and 21 months of age, respectively. (**A**) Representative images showing the distribution of Aβ plaques (6E10 antibody staining) in the hippocampus. Scale bar, 300 μm. (B–D) Quantification of Aβ plaques in the hippocampus. Vaccination with rCV02 significantly reduced the plaque count (**B**), percentage of brain area occupied by plaques (**C**), and Aβ load (**D**). Data represent the mean ± SD for each group (n = 8). Statistically significant differences were determined by Student’s *t*-test. **p < 0.01, ***p < 0.001, compared with the control group (3 × Tg-AD).

**Figure 5:**
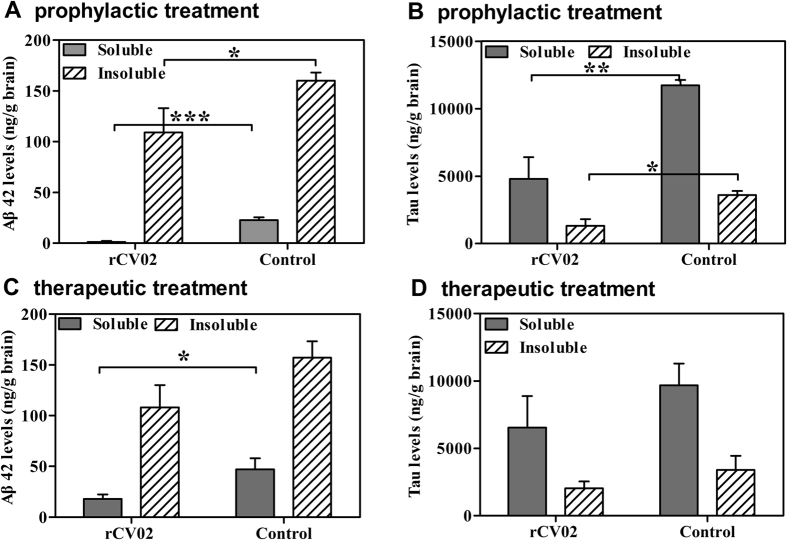
Vaccination with rCV02 reduced the level of Aβ42 and tau in the brains of 3 × Tg-AD mice. 3 × Tg-AD mice were immunized with rCV02 at 3 months (prophylactic; panels A and B) or 12 months (therapeutic; panels **C,D**) of age. The brains were collected for evaluation at 19 months and 21 months of age, respectively. (**A,C**) Soluble and insoluble Aβ42 levels in the brain were measured by sandwich ELISA. (**B,D**) Soluble and insoluble tau levels in the brain were measured by ELISA. Data represent the mean ± SE for each group (n = 8). Statistically significant differences were determined by Student’s *t*-test. *p < 0.05, **p < 0.01, ***p < 0.001, compared with the control group (3 × Tg-AD).

**Figure 6:**
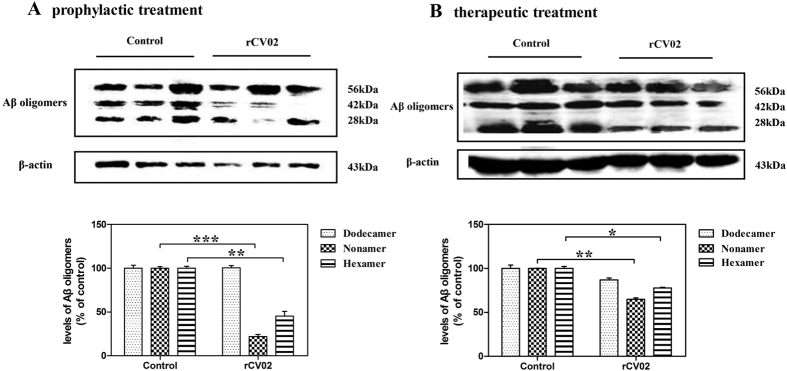
rCV02 reduced the levels of soluble Aβ oligomeric species in the brains of 3 × Tg-AD mice. 3 × Tg-AD mice were immunized with rCV02 at 3 months (prophylactic; panel **A**) or 12 months (therapeutic; panels **B**) of age. The brains were collected for evaluation at 19 months and 21 months of age, respectively. The 6-, 9- and 12-mers (28, 42 and 56 kDa) of Aβ in soluble fractions of brain homogenates were detected by Western blot analysis using the 6E10 monoclonal antibody; representative images are shown (lanes 1–3, non-vaccinated control 3 × Tg-AD; lanes 4–6, rCV02-vaccinated 3 × Tg-AD). Densitometry calculations were performed using Image J and normalized to β-actin. Data represent the mean ± SD for each group. Statistically significant differences were determined by Student’s *t*-test. *p < 0.05, **p < 0.01, compared with the control group (3 × Tg-AD).

To better understand the recovery of cognition in the rCV02-immunized mice, we sought to determine the molecular correlations associated with improvement. We first assessed whether rCV02 mitigated AD-like pathology in the brains of vaccinated 3 × Tg-AD mice (Figs 4, 5, 6). In both the prophylactic and therapeutic setting, immunization with rCV02 significantly reduced the number of visible plaques and the total percentage of the brain area occupied by plaques (Fig. 4A–C). Compared to the control AD mice, the Aβ plaque load in the hippocampus of the rCV02-immunized mice was reduced by 68.6% and 65.1% in the prophylactic and therapeutic models, respectively (Fig. 4D). Prophylactic treatment significantly reduced the levels of insoluble and soluble Aβ42 peptide in the brains of immunized mice compared to control mice (Fig. 5A). We also detected a significant reduction in soluble Aβ42 concentrations following therapeutic treatment (Fig. 5C).

The Aβ oligomeric species present in soluble fractions of brain homogenates of vaccinated and non-vaccinated 3 × Tg-AD mice were also detected by Western blot analysis. Levels of 6E10 monoclonal antibody-reactive Aβ oligomers, primarily hexamers and nonamers (28 and 42 kDa, respectively), were significantly reduced by prophylactic and therapeutic administration of rCV02 (Fig. 6, *p* < 0.05). Thus, Aβ oligomers appeared to decrease in immunized mice in parallel with changes in plaque load and cerebral Aβ levels.

Moreover, we assessed the tau pathology in the brains of immunized 3 × Tg-AD mice by immunohistochemistry and ELISA. A significant reduction in the number of HT-7 positive neurons of brains was observed in the brains of immunized mice compared to controls, which indicated that both prophylactic and therapeutic administration of rCV02 decreased total tau accumulation (*p* < 0.01, Supplementary Figure S2). The levels of insoluble and soluble total tau were also were significantly reduced in aged 3 × Tg-AD mice following prophylactic immunization (Fig. 5B), while it was no significantly reduced following therapeutic immunization (Fig. 5D). Thus, our results indicated that immunization with rCV02 markedly reduced the levels of Aβ and tau in the brains of aged 3 × Tg-AD mice, and improved behavioral performance.

### Immunization with rCV02 decreases calpain activation and prevents the loss of synaptic proteins in aged 3 × Tg-AD mice

**Figure 7:**
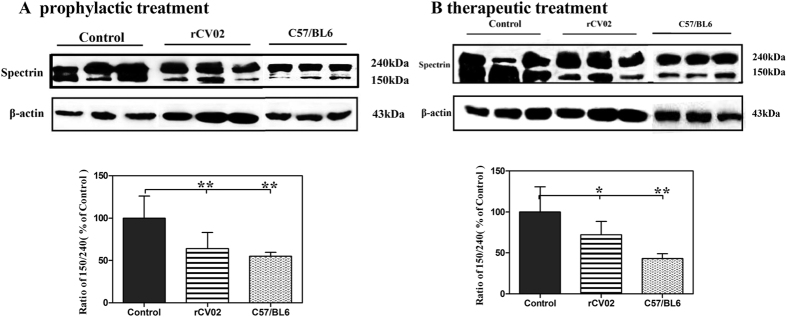
rCV02 decreased calpain activation in the brains of 3 × Tg-AD mice. 3 × Tg-AD mice were immunized with rCV02 at 3 months (prophylactic; panel **A**) or 12 months (therapeutic; panels **B**) of age. The brains were collected for evaluation at 19-months and 21-months of age, respectively. The spectrin content in the soluble fractions of the brain homogenates were detected by Western blot analysis; representative images are shown (lanes 1–3, non-vaccinated control 3 × Tg-AD; lanes 4–6, rCV01-vaccinated 3 × Tg-AD; lanes 6–9, non-vaccinated control C57/BL6). The graphs show the spectrin 150/240 kDa ratio in control and treated mice. Data represent the mean ± SE for each group. Statistically significant differences were determined by ANOVA. **p < 0.01, compared with the control group (3 × Tg-AD).

Aβ accumulation has been shown to induce calpain activation^26^,^27^,^28^,^29^,^30^, which may lead to the degradation of dynamin 1, a protein involved in synaptic vesicle release, and postsynaptic density protein PSD-95, which is crucial for synapse maturation and plasticity^31^,^32^,^33^. Given the remarkable recovery of cognition in the rCV02-immunized aged 3 × Tg-AD mice with reduced AD-like pathology, we further explored the molecular mechanisms associated with this improvement. We first determined whether administration of rCV02 decreased calpain activation in 3 × Tg-AD mice. Spectrin degradation is highly sensitive to calpain activation and considered an excellent marker of the activity of this protease^26^,^29^. Western blot analysis of the immunoreactivity of soluble fractions of brain homogenates with a specific spectrin antibody showed a significant increase in the spectrin 150/240 kDa ratio in the brains of aged 3 × Tg-AD mice compared to age-matched C57/BL6 mice. Prophylactic or therapeutic administration of rCV02 prevented the increase in the spectrin 150/240 ratio in aged 3 × Tg-AD mice (Fig. 7A,B), which indicated that immunization with rCV02 decreases calpain activation in aged 3 × Tg-AD mice.

**Figure 8:**
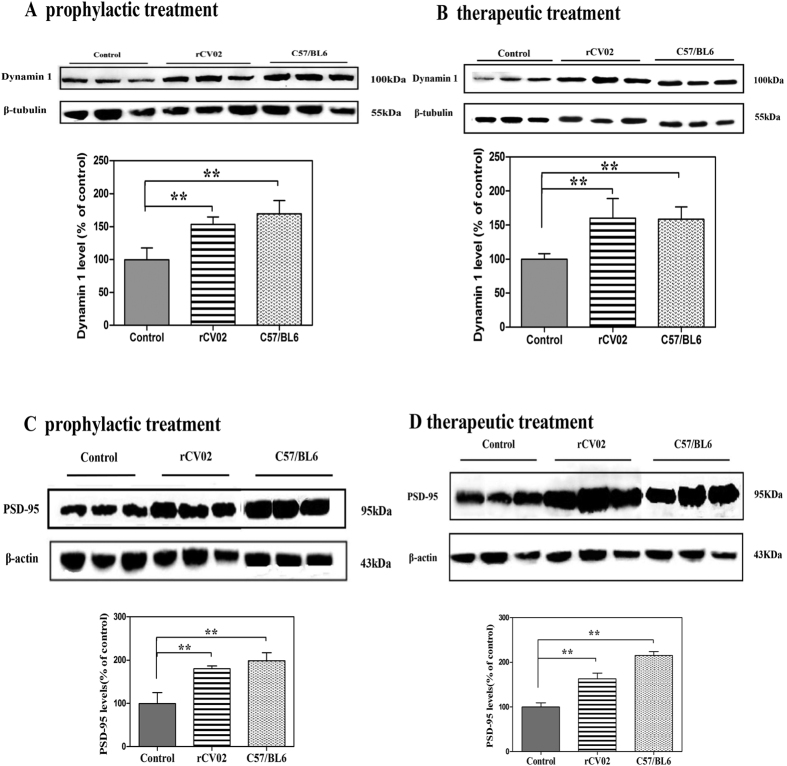
rCV02 increased the levels of synaptic proteins in the brains of 3 × Tg-AD mice. 3 × Tg-AD mice were immunized with rCV02 at 3 months (prophylactic; panel **A** and **C**) or 12 months (therapeutic; panels **B** and **D**) of age. The brains were collected for evaluation at 19 months and 21 months of age, respectively. Levels of dynamin 1 (**A,B**) and PSD-95 (**C,D**) in the soluble fractions of the brain homogenates were detected by Western blot analysis; representative images are shown (lanes 1–3, non-vaccinated control 3 × Tg-AD; lanes, 4–6 rCV01-vaccinated 3 × Tg-AD; lanes 6–9, non-vaccinated control C57/BL6). The graphs show the levels of dynamin 1 (A, B) and PSD-95 (**C,D**) in control and treated mice. Data represent the mean ± SE for each group. Statistically significant differences were determined by ANOVA. *p < 0.05, **p < 0.01, compared with the control group (3 × Tg-AD).

To examine the potential of rCV02 immunization to prevent the loss of synaptic proteins or to mitigate synaptic impairment, we assessed the levels of dynamin 1 and PSD-95 in the brains of mice. Compared to the age-matched C57/BL6 controls, the non-immunized 3 × Tg-AD mice had significantly reduced levels of both synaptic proteins in the brain. However, prophylactic or therapeutic administration of rCV02 prevented the significant decrease of dynamin 1 (Fig. 8A,B) and PSD-95 (Fig. 8C,D) in aged 3 × Tg-AD mice. Thus, along with the behavioral improvement, immunization with rCV02 significantly decreased calpain activation concurrent with reduced soluble Aβ or oligomeric forms of Aβ in aged 3 × Tg-AD mice. This effect may prevent dynamin 1 and PSD-95 degradation.

### Immune mechanism underlying the use of a tetanus toxin fragment (THc-C) of rCV02 as carrier protein

**Figure 9:**
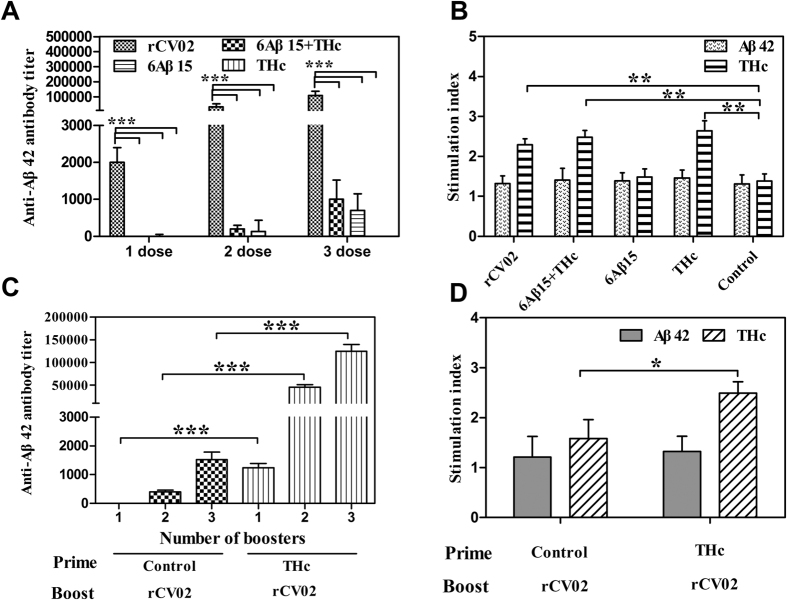
Immune mechanism underlying the effects of the tetanus toxin fragment (THc-C) of rCV02 as a carrier protein in 3 × Tg-AD mice. Titer of anti-Aβ42 antibodies (**A**) and T cell proliferation (**B**) were analyzed in 3 × Tg-AD mice immunized with different vaccines. The rCV02 vaccine induced strong Aβ-specific humoral immunity via help from foreign Th cells specific to the Th epitopes of THc-C. Titer of anti-Aβ42 antibodies (**C**) and T cell proliferation (**D**) were also analyzed in THc-immunized aged 3 × Tg-AD mice. Strong humoral and cellular immune responses to rCV02 were generated in THc-immunized aged 3 × Tg-AD mice. Data represent the mean ± SD (n = 8). Statistically significant differences were determined by ANOVA or Student’s *t*-test. * *p* < 0.05, **p < 0.01,***p < 0.001, compared with 6Aβ15 or the control group.

The novel recombinant 6Aβ15-THc-C chimeric vaccine (rCV02) was reconstructed by fusion of 6Aβ15 with THc-C, which had been proposed as a carrier or molecular adjuvant^34^,^35^. Here, the immune mechanism of THc-C of rCV02 as carrier protein was further defined in 3 × Tg-AD mice. As shown in Fig. 9A,B, immunization with rCV02 induced strong Aβ-specific humoral immune responses and activated THc-specific Th cell proliferation (*p* < 0.05, compared to 6Aβ15 or control groups), but did not activate Aβ-specific Th cells in mice. In contrast, immunization with 6Aβ15 or 6Aβ15 + THc induced only very mild Aβ-specific antibody responses (*p* < 0.001, compared to rCV02). Thus, the presence of the foreign toxin fragment as a carrier protein in the rCV02 vaccine was critical for generating strong Aβ-specific antibodies without activating self-reactive Aβ42-specific T cells.

We also evaluated the capacity of rCV02 to elicit a rapid and strong antibody response in THc-immunized old 3 × Tg-AD mice (Fig. 9C,D). Two or three boosters with rCV02 induced robust anti-Aβ antibody responses only in THc-immunized old 3 × Tg-AD mice with THc-specific pre-existing memory Th cells. Multiple immunizations with rCV02 still generated a relatively low antibody response in control mice. Moreover, a stronger Th cell response specific to THc was also detected in this group of mice with pre-existing memory Th cells compared with the responses generated in control mice (Fig. 9D, *p* < 0.05). Thus, these data indicated that immunization with rCV02 could activate pre-existing THc-specific memory Th cells, leading to rapid and strong THc-specific cellular responses and anti-Aβ antibody responses. Taken together, rCV02 induced strong Aβ-specific humoral immunity via the help provided by the foreign Th cells specific to the Th epitopes of THc-C and generated a rapid and strong Aβ-specific antibody response in aged 3 × Tg-AD mice with pre-existing memory Th cells.

## Discussion

The objective of this study was to undertake a comprehensive evaluation of the immunopotency of a novel clinical grade epitope immunogen 6Aβ15–THc-C formulated with alum adjuvant (rCV02) in 3 × Tg-AD mice and to determine the subsequent effects on Aβ-related pathologies and cognition. The recombinant chimeric 6Aβ15–THc-C immunogen was developed as a novel Aβ B-cell epitope candidate vaccine composed of hexavalent foldable Aβ1–15 fused with a tetanus toxin fragment (THc-C) as carrier protein or molecular adjuvant. This chimeric vaccine was designed to circumvent the problem of Aβ42-specific T cell autoreactivity and to overcome tolerance induction to self-antigens. In this study, immunization with rCV02 induced a strong Th2-polarized anti-Aβ antibody response in 3 × Tg-AD mice via the help of a toxin-derived carrier protein without the induction of autoimmune Aβ42-specific T cell responses. Both prophylactic and therapeutic administration of rCV02 mitigated Alzheimer’s disease-like pathology, cognitive decline and synaptic impairment in aged 3 × Tg-AD mice.

A potential problem of AD immunotherapy highlighted in previous studies is that a reduction of insoluble Aβ or Aβ deposits may lead to increased levels of the soluble forms of this peptide^36^, especially the most neurotoxic oligomers, which will impair synaptic and cognitive function^16^,^37^,^38^,^39^. In a previous study, the antibodies elicited by 6Aβ15-THc-C immunization bound to Aβ oligomers and inhibited Aβ42 oligomer-mediated neurotoxicity *in vitro*^25^. This indicated the potential of based-rCV02 vaccination to mediate therapeutic effects in 3 × Tg-AD mice *in vivo*. Here, we show that prophylactic immunization with rCV02 reduces not only Aβ plaques, but also soluble Aβ peptide, including Aβ nonamers and hexamers. These findings are consistent with those of previous reports^24^,^40^,^41^,^42^. The therapeutic effects of immunization with rCV02 in reducing the soluble Aβ are very important because the elicited anti-Aβ antibodies will clear or neutralize the oligomeric Aβ, producing beneficial cognitive effects.

Remarkably, in our study prophylactic immunization significantly reduced the total levels of insoluble and soluble tau in the brain in aged 3 × Tg-AD mice. While Aβ accumulation may be the primary event in AD pathogenesis and accelerate phosphorylation of tau or NFT formation^43^,^44^, tau pathology involved in synaptic loss also plays an important role in disease progression^12^,^45^,^46^. Previous studies have shown that Aβ immunotherapy decreases Aβ and tau pathology in 3 × Tg-AD mice^42^,^47^,^48^,^49^,^50^, which is consistent with the results presented here. Coincidentally, a decrease in soluble tau levels has been reported in the CSF of immunized patients^6^ and in the brain of immunized 3 × Tg-AD mice^49^. Therefore, our results suggest that the rCV02-mediated reduction in Aβ may facilitate the clearance of total tau-related pathology. Furthermore, previous reports indicate that a reduction in both soluble Aβ and tau is necessary or beneficial in synergistically ameliorating cognitive impairments^49^,^50^.

In AD, cognitive decline is associated with synapse loss, which precedes neuron death. The neurotoxic effects of Aβ on the central synapses have been described previously and are reflected in the decrease levels of some synaptic proteins^14^,^16^,^51^. Indeed, the levels of dynamin 1 and PSD-95 in unvaccinated 3 × Tg-AD mice were significantly reduced compared to those in age-matched non-transgenic controls (C57/BL6). It is well established in animal AD models that Aβ-induced synaptic injury or neuronal loss can be prevented by calpain inhibitors^28^,^29^ or Aβ-directed immunotherapy^17^,^19^,^20^,^24^. Therefore, we hypothesized that immunization with rCV02 could decrease calpain activation and further protect synaptic function. In the current study, calpain activation was significantly decreased in the brains of immunized-rCV02 3 × Tg-AD mice. Along with the decreased calpain activity and Aβ levels, there were obviously increased levels of dynamin 1 and PSD-95. Thus, it is likely that the decreased calpain activity contributed to preventing degradation of dynamin 1 and PSD-95. These results were suggestive of a remarkable association between the levels of Aβ, cognitive function, and synaptic function in 3 × Tg-AD mice. Taken together, these findings demonstrate that selective reduction of soluble Aβ or oligomers by immunotherapy with rCV02 is sufficient to prevent the loss of the synaptic proteins, allowing functional recovery of cognition. Since synaptic dysfunction is an important phenotypic manifestation of AD^14^,^20^,^22^,^38^, it can be speculated that there may be a molecular link to AD pathogenesis that will allow dynamin 1 and PSD-95 to be useful as biomarkers for evaluating prospective treatments for AD.

In the design of the rCV02 vaccine, we selected the immunodominant B-cell epitope peptide of Aβ42 as the immunogen and constructed a hexavalent foldable Aβ1–15, with each peptide sequence separated by a GS small linker (6Aβ15). This novel immunogen for AD was designed to mimic the assembly states of Aβ42 and improve immunogenicity over that of short Aβ1–15 peptide. We also replaced the T cell epitopes of Aβ42 with the C fragment of TeNT (THc-C) with the aim of activating non-self pre-existing memory Th cells in the general human population with a conventional tetanus toxoid (TT) vaccine. Data from this study demonstrate that immunization with rCV02 produced robust anti-Aβ42 antibody responses while breaking immune self-tolerance to the Aβ42 self-antigen in 3 × Tg-AD mice. It should be noted that the enhanced Aβ-specific antibody response was observed only when the 6Aβ15 antigen was fused directly to the THc-C carrier protein. Thus, it can be speculated that the strong immunogenicity of rCV02 can be attributed to the fusion construct, in which the THc-C carrier protein acts as an efficient molecular adjuvant. Moreover, in presence of THc-specific memory Th cells, rCV02 mediated strong activation of pre-existing memory Th cells specific to the Th epitopes of this vaccine and led to a rapid and robust production of antibodies specific to the B-cell epitope (Aβ1–15) of the same vaccine as previously reported^52^. Therefore, the immunogenic carrier protein THc-C in rCV02, containing strong universal CD4^+^T cell epitopes^34^,^35^, can provide T cell help and activate pre-existing THc-specific memory Th cells for Aβ-specific antibody production.

The prophylactic treatment of 3-month-old 3 × Tg-AD mice showed a trend toward the generation and maintenance of relatively stable and adequate antibody levels over a 7-month period after the last vaccination. The therapeutic treatment of 12-month-old 3 × Tg-AD mice generated a relatively low antibody response and there was an obvious reduction in Aβ-specific antibody levels after the last vaccination. Lower levels of Aβ and tau and greater cognitive benefits were observed in the prophylactic model, which is consistent with previous reports of the correlation of high titers of therapeutic anti-Aβ antibodies with reduced Aβ pathology and improved cognitive ability in preclinical and clinical trials^6^,^8^. The post-vaccination production of Aβ-specific antibodies over time may be considered a pharmacological response of the immune system to the vaccine. The generation of robust immunity was more difficult due to old age-associated hyporesponsiveness in elderly 3 × Tg-AD mice. However, rCV02 immunization overcame hyporesponsiveness in 12-month-old 3 × Tg-AD mice with THc-specific memory T cells and generated a rapid and strong Aβ-specific humoral response. Therefore, the immunological mechanism of action of rCV02 indicate this vaccine would be highly beneficial for inducing therapeutically potent anti-Aβ antibody responses in future clinical trials of middle-aged or elderly AD patients with pre-existing memory.

To avoid stimulating adverse immune responses, second-generation AD vaccines targeting the N-terminal epitopes of Aβ-specific B cells have been developed and are being tested in current clinical trials^5^,^8^,^12^. In addition, universal CD4^+^ T cell epitopes have been identified within the C fragment of TeNT and other proteins, and their use as carrier proteins has been tested in both mice and humans^35^,^53^. Elan and Wyeth designed an AD vaccine ACC-001, in which an N-terminal sequence of Aβ (Aβ1–6) was conjugated to diphtheria toxin (DT) to provide foreign Th epitopes to overcome Aβ-associated hyporesponsiveness for Aβ-specific antibody production. MER5101 is also a similar Aβ1–15:DT conjugate vaccine for AD^54^. A recombinant protein vaccine (Lu AF20513) composed of two foreign Th cell epitopes from TT, P30, and P2 and three copies of the B-cell epitopes of Aβ42 (Aβ1–12) is currently being tested in phase I trials^52^. Lu AF20513 is expressed in *E. coli* and is found primarily in the inclusion bodies, while in this study a novel recombinant chimeric 6Aβ15-THc-C antigen expressed in *E. coli* (BL21) in a fully soluble form was constructed and developed as the rCV02 vaccine for AD. Unlike the current ACC-001 or other vaccines in which an N-terminal Aβ sequence is conjugated to DT or other carriers, this type of recombinant protein vaccine carries the advantages of expected safety as well as ease of construction and large-scale production in a chemically homogeneous form. Moreover, these two recombinant protein vaccines may represent an effective and safe form of active immunotherapy that may overcome the Aβ and old age-associated hyporesponsiveness via the help of foreign Th cell epitopes from TT^52^.

In summary, we have comprehensively characterized the immunogenicity, efficacy, and mechanism of action of rCV02 in both prophylactic and therapeutic 3 × Tg-AD mouse models. Our findings indicate the promise of rCV02 as a novel candidate vaccine for AD as well as the potential for inducing potent anti-Aβ antibody responses in AD patients with pre-existing memory Th cells specific to TT. After completion of preclinical safety and toxicity studies, human clinical trials of the rCV02 as a new prophylactic vaccine for AD will be initiated.

## Methods

### Preparation of rCV02

In this study, a novel recombinant chimeric 6Aβ15-THc-C immunogen was expressed in *Escherichia coli* (BL21) in a fully soluble form^25^ and purified through a series of chromatographic methods and buffer exchanges to yield the final formulation. We used the recombinant chimeric 6Aβ15-THc-C immunogen as a novel Aβ B-cell epitope vaccine (rCV02) containing a hexavalent foldable Aβ1–15 (6Aβ15) fused to a tetanus toxin fragment (THc-C) as a carrier protein or molecular adjuvant. The vaccine for AD was formulated in a Th2-biased aluminum hydroxide adjuvant (Brenntag Biosector, Frederikssund, Denmark).

### Immunization of 3 × Tg-AD mice with rCV02

A colony of 3 × Tg-AD homozygous mice harboring the human APP_Swe_, PS1_M146V_, and Tau_P301L_ mutations were generated from breeding pairs obtained from Jackson Lab (Bar Harbor, ME, USA). All 3 × Tg-AD mice were housed in a temperature- and light-cycle controlled animal facility at the Beijing Laboratory Animal Center (Beijing, China). All animal procedures were conducted with the approval of the Beijing Institute of Biotechnology Institutional Animal Care and Use Committee and the methods were carried out in accordance with the approved guidelines. Eight 3 × Tg-AD mice (aged 2 months, 4 females and 4 males) were immunized with 5 μg purified 6Aβ15-THc-C immunogen formulated with 0.2% (w/w) Alhydrogel^TM^ (Brenntag Biosector). Four injections were administered at 2-wk intervals (100 μl/injection). PBS formulated with the adjuvant was used as a negative control.

To assess the protective effects of rCV02 in 3 × Tg-AD mice, we established prophylactic (Supplementary Fig. S1A) and therapeutic (Supplementary Fig. S1B) models. The prophylactic group consisted of 8 mice (4 females and 4 males) that were aged 3 months at the time of immunization and did not have obvious behavioral impairment or amyloid plaques. The prophylactic group was immunized intramuscularly (i.m.) four times with 5 μg of 6 Aβ15-THc-C (rCV02) at 1-month intervals, with a final booster immunization administered 5 months after the fourth dose. The therapeutic group consisted of 8 mice (4 females and 4 males) that were aged 12 months at the time of immunization and had the typical behavioral impairment and amyloid plaque deposition. The therapeutic group was immunized i.m. three times with rCV02 at 1-month intervals, and received two booster immunization (the first 2 months after the third dose and the second 3 months later). Eight 3 × Tg-AD mice (4 females and 4 males) receiving only vehicle was used as a negative control.

We performed two experiments in 3 × Tg-AD mice to further assess the mechanism of the immune response associated with rCV02 vaccination. In one experiment, groups of eight mice (aged 3 months) were immunized three times at 1-month intervals with 5 μg of 6 Aβ15-THc-C (rCV02), 6Aβ15^25^, THc of tetanus toxin^55^, or 6Aβ15 + THc (a mixed vaccine composed of 6Aβ15 and THc) formulated with Alhydrogel^TM^ 0.2% (w/w). In another experiment, 3- month-old mice were prime-immunized twice at 1-month intervals with 5 μg of THc formulated with 0.2% (w/w) Alhydrogel^TM^. Control mice received the aluminum hydroxide adjuvant only. The 12-month-old mice from the THc-primed and control groups were boosted with 1, 2 or 3 doses of rCV02 at 1-month intervals after a resting period of 8 months. The humoral and cellular immune responses were analyzed in experimental and control mice.

### Detection of antibody responses

ELISAs were used to determine the levels of anti-Aβ42 antibodies in sera from mice in the different treatment groups as previously described^25^. Briefly, the sera were serially diluted (from 100-fold) before testing. The total IgG and the IgG isotype antibody responses were detected using horseradish peroxidase (HRP)-conjugated anti-mouse antibodies (Santa Cruz Biotechnology, Inc., Santa Cruz, CA, USA) at a dilution of 1:2, 000. After incubation with the secondary antibody, reactivity was visualized by adding 100 μl of citrate buffer (pH 5.0) containing 0.04% (w/v) o-phenylenediamine and 0.02% (v/v) hydrogen peroxide for 10 min at room temperature. The reaction was stopped by the addition of 50 μl of 2 M H_2_SO4, and the absorbance was read at 492 nm using a Thermo Labsystems microplate reader (Franklin, MA, USA). Serum samples from individual mice were assayed, and the geometric mean titer (GMT) was calculated for each group (n = 8).

### Lymphocyte proliferative responses and evaluation of cytokine levels

Cell suspensions of splenocytes from immunized mice were prepared and treated as previously described^25^. The proliferative responses of splenocytes were determined using the cell counting kit-8 (CCK-8; Dojindo Molecular Technologies, Kumanoto, Japan) according to the manufacturer’s protocol. The levels of mouse IL-4 and IFN-γ produced by the splenocytes were assayed using commercial ELISA kits according to the manufacturer’s protocol (Bender Medsystems, Vienna, Austria). Data are presented as means ± SD of n = 8 samples.

### Behavioral test

The Morris water maze was used for behavioral tests as previously described^25^. Briefly, the 3 × Tg-AD mice aged 19 months and 21 months that had received five vaccinations were trained to swim in the Morris water maze apparatus. Age-matched non-transgenic C57/BL6 mice were used as controls (n = 6, 3 females and 3 males). For each trial, the mouse was released into the tank at one of four designated start locations and allowed to find and escape onto the hidden platform. During the learning period each mouse was subjected to a daily four-trial session for seven consecutive days. The escape latency was recorded as the time from being put into the water to climbing the escape platform during training. Retention (probe trial) of spatial learning was assessed 24 h after the last training trial. The platform was removed from the pool, and each mouse was subjected to one 60 s swim probe trial. The parameters measured during the probe trial were the initial latency to reach the platform location and number of platform location crosses.

### Immunohistochemistry and semi-quantitative image analysis

After the Morris water maze test, brains were removed from the 3 × Tg-AD mice aged 19 months and 21 months and divided sagittally along the interhemispheric fissure; half of the brain was used for immunohistochemical analysis. Immunohistochemical analysis and quantification of the stained sections were conducted as previously described^25^,^56^. The Aβ deposits were detected using the anti-human Aβ monoclonal antibody 6E10 (1:1,000, Covance, Emeryville, CA, USA) and general tau pathology was detected with HT7 (1:40, Thermo scientific, Waltham, MA, USA), recognizing epitopes 159–163.

### Determination of Aβ and tau levels in soluble/insoluble fractions

The other half of the brain collected following the Morris water maze test was homogenized in Tris-buffered saline (TBS) buffer containing a protease inhibitor cocktail (Roche, Mannheim, Germany). The samples were ultrasonicated and then centrifuged at 100, 000 × *g* for 2 h at 4 °C. The supernatant was collected to detect soluble Aβ42 or tau. The sediment was treated with 5 M guanidine buffer to solubilize the Aβ or tau. Biosource ELISA kits (Invitrogen, Carlsbad, CA, USA) were used to detect Aβ42 and total tau levels in the brain extracts. Aβ levels were calculated as ng/g brain (wet weight).

### Western blot analysis

Western blot analyses were performed to determine the levels of Aβ oligomers, dynamin 1, spectrin, and PSD-95 in the soluble fractions of the brain homogenates from immunized, control (3 × Tg-AD), and wild-type (C57/BL6) mice. Protein samples in TBS buffer were separated by SDS-PAGE (10–12% gels), and transferred onto a PVDF membrane. The membrane was blocked with 10% non-fat milk in 20 mM Tris-HCl (pH 7.4) containing 150 mM NaCl and 0.05% Tween 20 (TBS-T) After washing with TBS-T, the membrane was incubated with the following primary antibodies: anti-human Aβ monoclonal antibody 6E10 (1:1,500), anti-dynamin 1 (C16, 1:2,000, Santa Cruz Biotechnology, Inc.), anti-spectrin (AA6, 1:1,500, Millipore, MA, USA), or anti-PSD-95 (1:500, Invitrogen), or anti-β-actin/tubulin (Sigma, MO, USA) for 1 h at 37 °C. The membrane was incubated with a HRP-conjugated IgG (GBI, WA, USA) secondary detection antibody (1: 5,000) for 0.5 h at 37 °C and protein immunoreactivity was visualized using enhanced chemiluminescence reagents (ECL, Pierce, IL, USA). Proteins were quantified by densitometry using the Image J Software and normalized to β-actin/tubulin. The values obtained in untreated controls were considered to represent 100% expression. Values represent the means ± SE for each group (n = 6–8).

### Statistical analysis

All statistical analyses were performed using the SPSS software (version 16.0, Chicago, IL, USA). Statistically significant differences between groups were determined using the one-way analysis of variance (ANOVA) or Student’s *t*-test. For all tests, *P*-values < 0.05 were considered to indicate statistical significance.

## Additional Information

**How to cite this article**: Yu, Y.-Z. *et al*. A novel recombinant 6Aβ15-THc-C chimeric vaccine (rCV02) mitigates Alzheimer’s disease-like pathology, cognitive decline and synaptic loss in aged 3 × Tg-AD mice. *Sci. Rep.* **6**, 27175; doi: 10.1038/srep27175 (2016).

## Supplementary Material

Supplementary Information
