# Supplementary material for: A novel recombinant 6Aβ15-THc-C chimeric vaccine (rCV02) mitigates Alzheimer’s disease-like pathology, cognitive decline and synaptic loss in aged 3 × Tg-AD mice
Source: Sci Rep. 2016 Jun 3;6:27175. doi: 10.1038/srep27175 (PMC4891678; doi:10.1038/srep27175)
Supplement: Supplementary Information [file srep27175-s1.pdf]

A novel recombinant 6Aβ15-THc-C chimeric vaccine (rCV02) mitigates Alzheimer's disease-like pathology, cognitive decline and synaptic loss in aged 3×Tg-AD mice

Yun-Zhou Yu, Si Liu, Hai-Chao Wang, Dan-Yang Shi, Qing Xu, Xiao-Wei Zhou,

Zhi-Wei Sun, Pei-Tang Huang

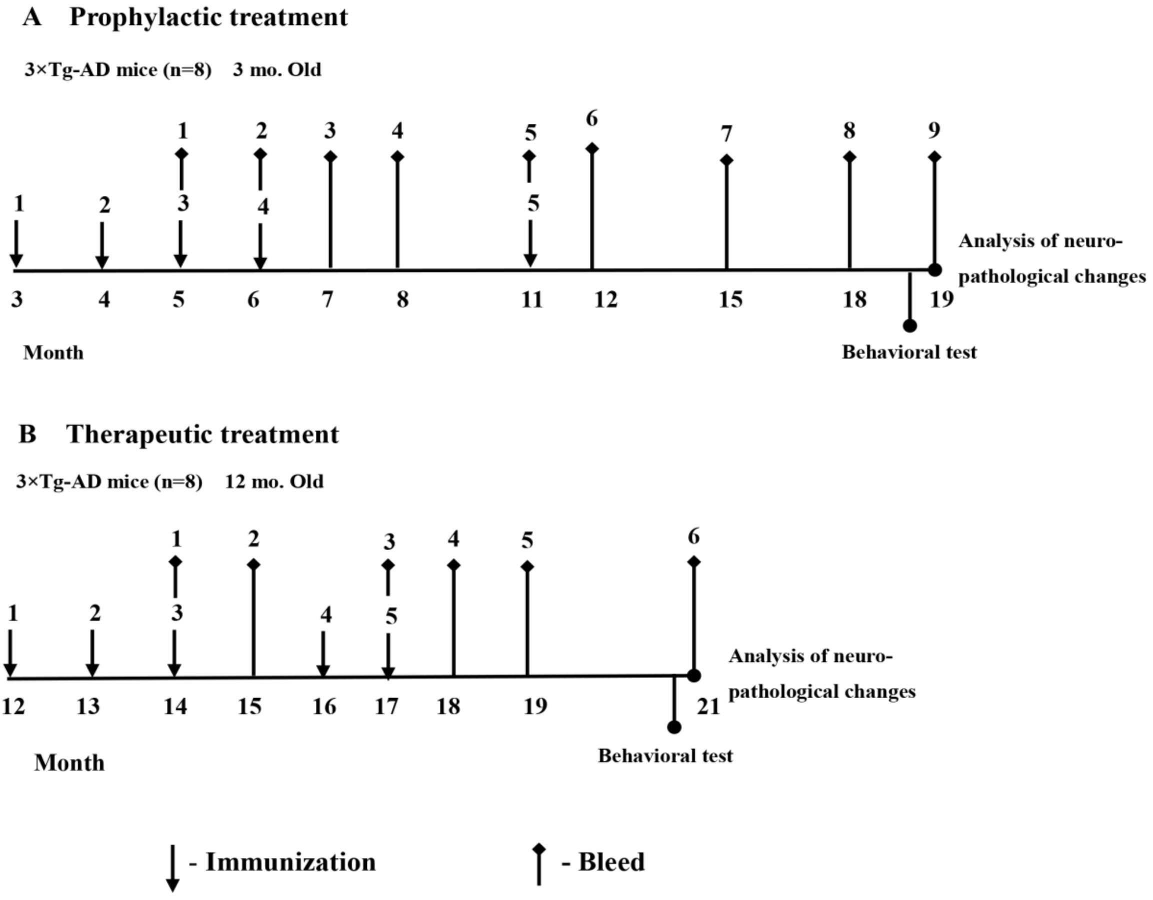

Supplementary Figure S1 Experimental designs of the prophylactic and therapeutic administration models.

All of the 3×Tg-AD mice were immunized five times with 5 μg of 6Aβ15-THc-C antigen formulated with 0.2% (w/w) Alhydrogel. (A) In the prophylactic treatment the mice were immunized intramuscularly four times with rCV02 at 1-month intervals. The booster (5th dose) was administered 5 months after the last monthly dose. (B) In the therapeutic group, the mice were immunized i.m. three times with rCV02 at 1-month intervals (doses 1–3). Two boosters were administered; the first two months after dose 3 (4th dose), and the second one month after dose 4 (5th dose). Blood was collected as indicated and serum antibody levels were analyzed. After the 5th immunization, behavioral studies were conducted (19 or 21 months, respectively). Following completion of the Morris water maze testing, the mice were sacrificed and the brain was analyzed for neuropathological changes.

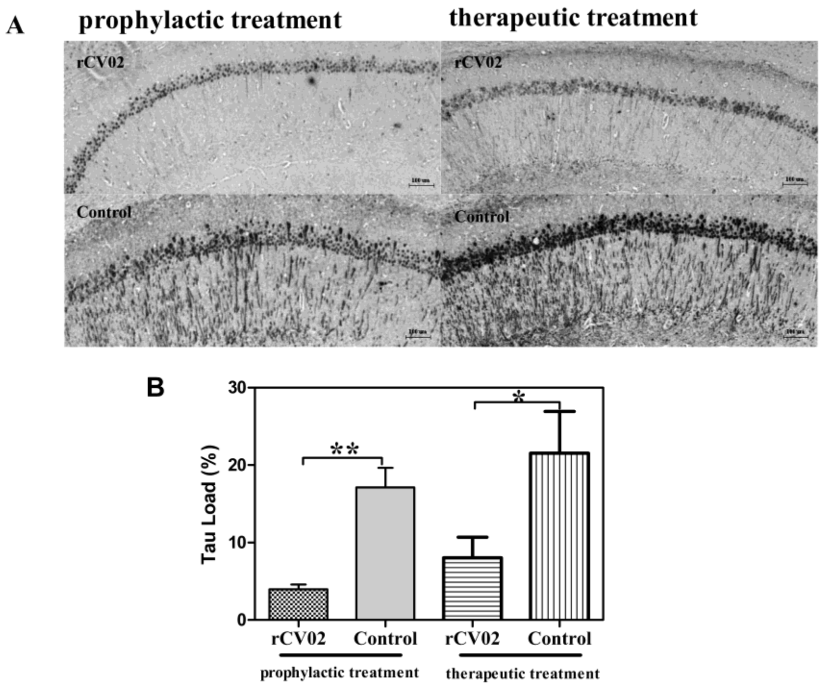

Supplementary Figure S2 rCV02 decreased the total tau levels in the brains of 3×Tg-AD mice.

3×Tg-AD mice were immunized with rCV02 at 3 months (prophylactic treatment) or 12 months (therapeutic treatment) of age. The brains were collected for evaluation at 19 months and 21 months of age, respectively. (A) Representative images of immunohistochemical staining for tau^+ areas (HT-7 antibody) in the hippocampus of brain. Scale bar, 100 μm. (B) Quantification of total tau (HT-7) load in the hippocampus of brains. Data represent the mean ± SD for each group (n=8). Statistically significant differences were determined by Student's *t*-test. *$P < 0.05$, **$P < 0.01$, compared with the control group (3×Tg-AD).
